# Disruption and pseudoautosomal localization of the major histocompatibility complex in monotremes

**DOI:** 10.1186/gb-2007-8-8-r175

**Published:** 2007-08-29

**Authors:** Juliane C Dohm, Enkhjargal Tsend-Ayush, Richard Reinhardt, Frank Grützner, Heinz Himmelbauer

**Affiliations:** 1 Max Planck Institute for Molecular Genetics, Ihnestr. 63-73, 14195 Berlin, Germany; 2 School of Molecular and Biomedical Science, The University of Adelaide, Adelaide 5005 SA, Australia

## Abstract

**Background:**

The monotremes, represented by the duck-billed platypus and the echidnas, are the most divergent species within mammals, featuring a flamboyant mix of reptilian, mammalian and specialized characteristics. To understand the evolution of the mammalian major histocompatibility complex (MHC), the analysis of the monotreme genome is vital.

**Results:**

We characterized several MHC containing bacterial artificial chromosome clones from platypus (*Ornithorhynchus anatinus*) and the short-beaked echidna (*Tachyglossus aculeatus*) and mapped them onto chromosomes. We discovered that the MHC of monotremes is not contiguous and locates within pseudoautosomal regions of two pairs of their sex chromosomes. The analysis revealed an MHC core region with class I and class II genes on platypus and echidna X3/Y3. Echidna X4/Y4 and platypus Y4/X5 showed synteny to the human distal class III region and beyond. We discovered an intron-containing class I pseudogene on platypus Y4/X5 at a genomic location equivalent to the human HLA-B,C region, suggesting ancestral synteny of the monotreme MHC. Analysis of male meioses from platypus and echidna showed that MHC chromosomes occupy different positions in the meiotic chains of either species.

**Conclusion:**

Molecular and cytogenetic analyses reveal new insights into the evolution of the mammalian MHC and the multiple sex chromosome system of monotremes. In addition, our data establish the first homology link between chicken microchromosomes and the smallest chromosomes in the monotreme karyotype. Our results further suggest that segments of the monotreme MHC that now reside on separate chromosomes must once have been syntenic and that the complex sex chromosome system of monotremes is dynamic and still evolving.

## Abstract 2

The characterization and chromosomal mapping of major histocompatibility complex (MHC)-containing BAC clones from platypus and the short-beaked echidna reveals new insights into the evolution of both the mammalian MHC and monotreme sex chromosomes.

## Background

The major histocompatibility complex (MHC) is of central importance for adaptive and innate immunity in vertebrates [1]. Sequencing MHCs from several species of eutherian mammals and human has led to the identification of approximately 220 genes located within an interval of 3.5-4 Mbp [2,3]. The MHC region contains genes encoding class I and class II receptors that are involved in peptide display, genes that are responsible for peptide generation and transport, as well as genes encoding complement factors or cytokines. Many other genes with functions that are not related to immunity and defense are also located within the MHC.

**Figure 1:**
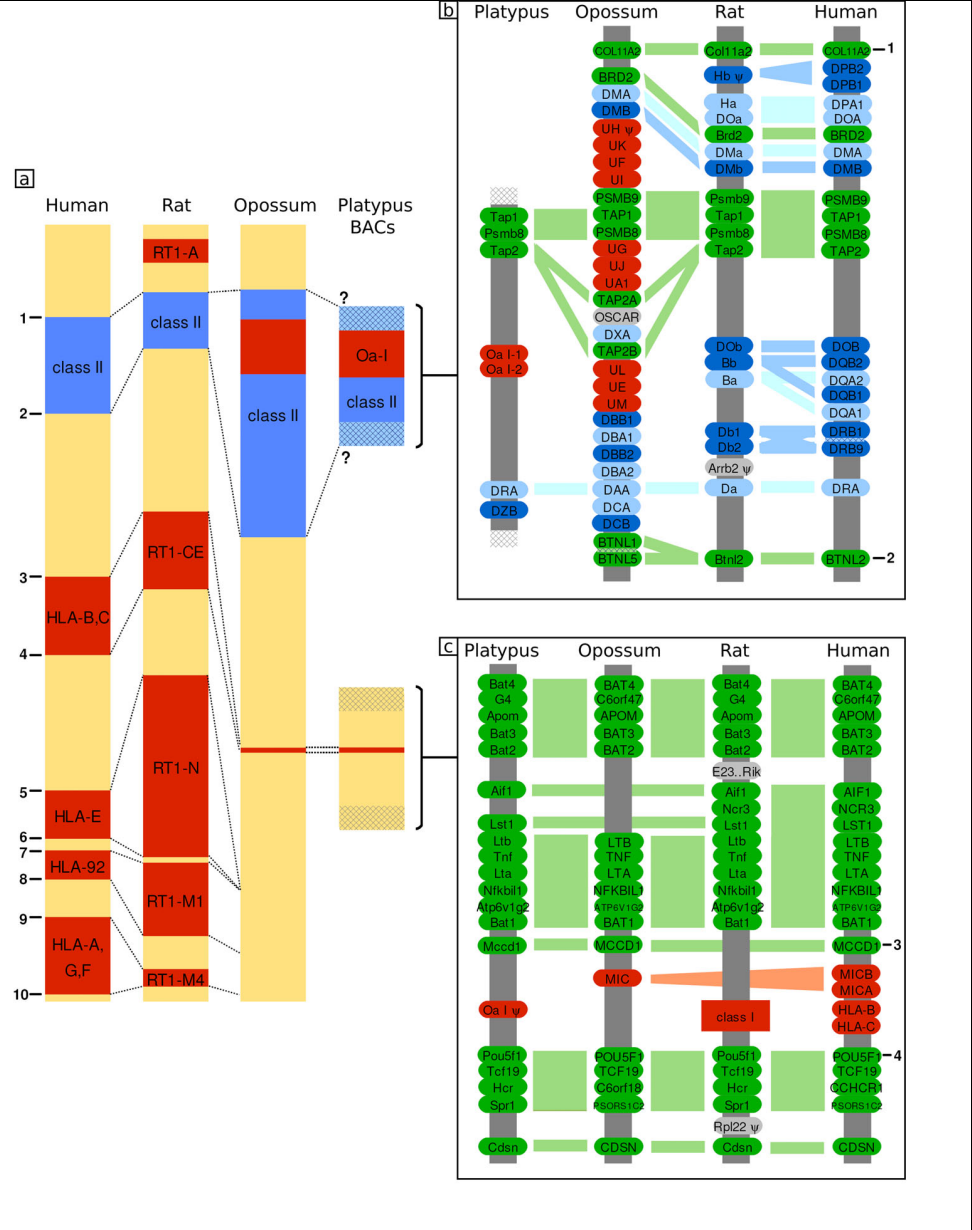
Comparative map of the mammalian MHC region. **(a) **Aligned MHC regions of human, rat, opossum [4,5,10] and the sequenced portions of the platypus MHC. Intervals are color-coded: class I regions are shown in red; class II regions in blue; and framework regions including class III in yellow. Circled numbers denote framework genes that typically flank these intervals: 1, *Col11a2*; 2, *Btnl2*; 3, *Bat1*/*Mccd1*; 4, *Pou5f1*; 5, *Gnl1*; 6, *Flj22638*; 7, *Trim39*; 8, *Trim26*; 9, *Tctex4*; 10, *Mog*. Dotted lines link orthologous positions defined by genes 1-10 in the four species. *Tctex4 *is missing from the MHC of opossum. Question marks indicate that the borders of the class I/II region in platypus have not been cloned. Rat has three additional RT1-M gene blocks outside the interval shown. **(b) **Comparison of the class II region of rat and human, and the opossum class I/II region to platypus based on annotation of platypus BAC 462c1. Genes are color-coded: framework genes are shown in green; class I genes in red; class II α chains in light blue; and class II β chains in dark blue (for phylogenetic relations among class II genes, see Additional data file 2). Non-class I and non-class II genes or pseudogenes unique for a given species are displayed in grey. Hatching between *BTNL2 *and *BTNL5 *in opossum and between *DRB1 *and *DRB9 *in the HLA indicates that further BTNL or DRB genes are located within these intervals. Orthologous genes are linked by colored bars. **(c) **Detailed map of the *Bat4 *to *Cdsn *interval, including a class I gene block.

The eutherian MHC subdivides into a single class II region followed by several class I regions, interlaced by framework gene regions. The central framework gene region, located between the class II region and the class I region that encodes the HLA-B and HLA-C genes in human, is also known as the class III region (flanked by the genes *Btnl2 *and *Bat1*/*Mccd1*; Figure 1a). This genomic architecture of MHC regions is remarkably well conserved in eutherian mammals. For instance, the position of the human HLA-B,C class I gene cluster matches rat RT1-CE, mouse H2-D,L,Q, swine SLA-6,7,8 and ovine β [4-8]. In each of these species, this particular class I gene block is flanked by the *Bat1*/*Mccd1 *and *Pou5f1 *framework genes. The same is true for the three other human class I gene clusters and their counterparts in other eutherian genomes, all of which locate at the same position, that is, are flanked by the same framework genes (see framework gene numbering in Figure 1a), even though not all of them contain active class I gene loci. The fact that only certain positions of the MHC support 'homing' of class I genes has previously been recognized and is known as the framework hypothesis [9].

In contrast to the conserved MHC architecture, much plasticity is observed within class I gene containing segments and, to a lesser extent, in the class II region. Orthologous class I genes are found in closely related species, for example, human and chimpanzees. Class I gene orthology becomes less apparent when more distantly related mammals are compared. This is attributed to the ongoing process of new class I genes arising by gene duplication and, at the same time, their disappearance from the genome by mutational inactivation or by deletion. For example, the sequences of the classical class I genes from rat form a separate clade when compared to class I genes from mouse, indicating species-specific evolution since separation of the two lineages 20 million years (Myr) ago [4].

In addition to comparative studies on eutherian mammals, other species have been studied and compared to the eutherians: recently, the sequence of a metatherian mammal (marsupial) MHC from the opossum (*Monodelphis domestica*) was assembled, based on whole genome shotgun sequence data [10]. Its annotation revealed a core MHC region, resembling the eutherian class II region, that, unlike in eutherian mammals, also encodes the class I genes. The opossum MHC was found to be syntenic to a human MHC region of several megabase pairs. Also in *Xenopus tropicalis *extensive synteny with the eutherian MHC has been found. Ohta *et al*. [11] analyzed the scaffolds that were generated from assembling *Xenopus *whole genome shotgun data for the presence of MHC-encoded genes, including framework genes. They found eight 200-900 kbp scaffolds that contained orthologs to many of the genes in the eutherian MHC. The interpretation is that the syntenies of the MHC region are ancient and predate the amphibian-mammalian split 350 Myr ago even though it has not been shown that these scaffolds reside on a single chromosome. In contrast, the MHC of the chicken, representing a lineage that is closer to mammals than *Xenopus *(distance aves-mammalia of 310 Myr [12]), is highly derived and does not align well with the human MHC [13]. The chicken MHC is remarkably different and contains only 19 genes within 92 kbp on chicken chromosome 16.

Monotremes are prototherian mammals, possessing fur and mammary glands, but uniquely combining reptilian features, for example, egg-laying with mammalian characteristics. They are the earliest offshoot of the mammalian clade that separated from theria (consisting of metatheria and eutheria, or marsupials and placental mammals, respectively) over 160 Myr ago [14]. The only extant prototheria are the duck-billed platypus (*Ornithorhynchus anatinus*), the short-beaked echidna (*Tachyglossus aculeatus*) and three species of long-beaked echidnas (*Zaglossus *sp.) [15]. The geographic range of current monotremes is restricted to Australia and nearby islands, including Papua New Guinea. In the following, when mentioning echidna, we refer to the short-beaked echidna, also known as the spiny anteater. 

Because of their unique phylogenetic position, monotremes are ideal species to unravel the characteristics of the ancestral mammalian MHC. So far, little is known about MHC genes in monotremes and there are only two reports where individual cDNA clones were identified as MHC class I or class II genes [16,17].

The general importance of monotremes for understanding the evolution and function of the mammalian genome is now generally accepted and is reflected by the presently ongoing efforts to sequence the platypus genome, with funding from the National Institute of Health (NIH) [18].

The platypus karyotype consists of 21 pairs of autosomes and 10 sex chromosomes (X1Y1-X5Y5 in males and X1X1-X5X5 in females). In addition, the platypus karyotype contains a number of very small chromosomes that have been proposed to be bird-like microchromosomes; however, the homology between these chromosomes and chicken microchromosomes has been controversial and no homologies have been identified to date [19,20]. The sex chromosomes assemble as a chain at prophase I during male meiosis where they adopt an XY alternating pattern, which ensures the segregation into X1-X5 and Y1-Y5 bearing sperm [21,22]. Although XY shared regions are required to ensure meiotic pairing, chromosome painting could not identify pseudoautosomal regions on some of the sex chromosomes so far. The echidna karyotype contains 27 autosomes and 10 sex chromosomes in females and 9 sex chromosomes in males, which where determined by counting the number of elements of the meiotic chain [23,24]. Very little is known about the gene content of both sex chromosome systems.

Here we describe the identification and characterization of genomic clones encompassing parts of the monotreme MHC. The unexpected localization of MHC genes on sex chromosomes of platypus and echidna provides valuable information about chromosomal homologies between aves, monotremes and eutheria, as well as homologies between platypus and echidna sex chromosomes.

## Results

### Identification of BAC clones from the platypus and echidna MHC

We identified bacterial artificial chromosome (BAC) clones from the platypus MHC by hybridization with oligonucleotide probes designed from the platypus class I gene Oran2-1 [16], the class II gene *DZB *[17], and from the MHC framework genes *Bat1 *and *Pou5f1 *encoding a DEAD-Box helicase and a stem cell transcription factor, respectively. Probes for the framework genes were designed by aligning the chicken *Bat1 *and *Pou5f1 *mRNA sequences with the rat MHC [4]. A 36-mer oligonucleotide probe for *Bat1 *corresponded to rat BAT1 amino acid residues 265-276, which are 100% conserved between rat, chicken and zebrafish. A 37-mer oligonucleotide probe was generated matching a segment of *Pou5f1 *whose translation had 100% amino acid identity in rat, chicken and zebrafish (rat POU5F1 residues 174-185).

**Figure 2:**
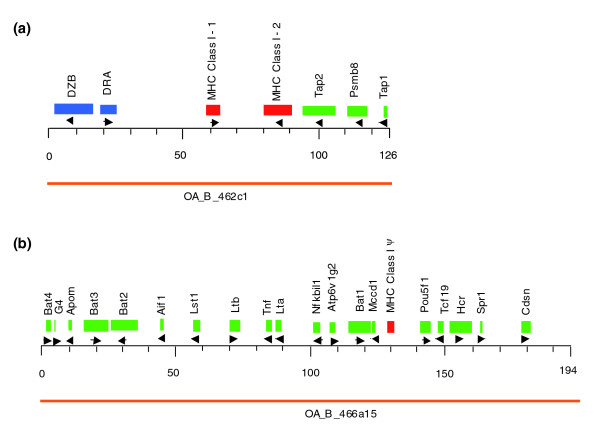
Platypus BACs and their gene content. (a) BAC clone 462c1, referred to as "BAC1" in the Results section (b) BAC 466a15 ("BAC2" in Results). Colors represent types of genes. Red: Class I genes; Blue: Class II genes; Green: Framework genes. Transcriptional orientation of genes is indicated by arrows.

Twenty-four platypus BACs positive with the class I probe and ten clones positive with the class II probe were identified in a BAC library with eleven-fold genome coverage. Based on Southern blots, two clones (BACs 446c3 and 462c1) were positive with both class I and class II gene probes (data not shown). More than 30 BAC clones were identified with the *Bat1 *and *Pou5f1 *probes. These genes co-localized in platypus, as several BACs hybridized with both probes. We sequenced and annotated the following three platypus BAC clones: BAC 462c1 (GenBank EU030443, class I and class II positive; Figure 2a); BAC 466a15 (GenBank EU030444, containing *Bat1 *and *Pou5f1*; Figure 2b); and BAC 362a17 (GenBank EU030442, class I positive). In the following, these BACs will be referred to as platypus BAC1, BAC2, and BAC3.

Using probes based on platypus MHC sequences, we searched for BACs from the corresponding regions in the echidna genome. A platypus *Tap2 *probe identified two positive clones in echidna (BACs 48g5 and 287o10; Additional data file 1a). Separate Southern blots prepared from these clones were hybridized with the Oran2-1 probe [16] and with a pool of two different class II gene oligonucleotide probes (Additional data file 1b,c). Echidna BAC 48g5 was found to contain class I and class II gene sequences, while BAC 287o10 contained class I sequences but no class II genes. Shared restriction fragments indicated that these two clones were from the same locus and overlapped.

Further echidna BAC colony screens were done using oligonucleotide probes designed on the platypus genes *Bat1 *and *Pou5f1*. For each gene, three probes were synthesized and pooled before labeling (one pool per gene). Echidna BAC clones 107j4, 129j16, 152g23 and 268a21 could be confirmed on Southern blots to contain both the *Bat1 *and *Pou5f1 *genes (Additional data file 1d,e).

### BAC sequence annotation

We generated reference gene models for the genes identified within platypus BACs 1-3, combining the gene prediction programs Genscan [25] and Genewise [26] as tools and utilized rat MHC protein sequences [4] as input for Genewise predictions. The translation products from predictions with either program were compared to each other and to the rat protein as a reference by pairwise blast (Additional data file 7). In the absence of monotreme expressed sequence tag (EST) sequences for the majority of genes, the annotations covered open reading frames (ORFs) and did not include untranslated regions (UTRs). Genscan and Genewise complement each other: The accuracy of Genewise is very good in coding regions that are well conserved, while theoretical gene models predicted by Genscan are exclusively based on signals and content. In total, we annotated 25 genes and two pseudogenes in the three BAC sequences. Within 320,837 sequenced bases in BAC1 and BAC2, one gene could be annotated with full ORF plus UTRs (*DZB *gene), 20 genes were annotated with their full ORFs only, two genes had missing 5' ends because of low conservation and two genes were only partially contained in the sequenced interval. One annotated feature was a non-processed pseudogene fragment that lacked an intact ORF, though partial reconstruction of the protein sequence was possible. Only one single gene feature was discovered and annotated as a pseudogene within BAC3 (see below).

### Characterization of a processed class I pseudogene containing platypus BAC clone

Platypus BAC3 was assembled to a finished sequence of 146,150 bp. Fluorescence *in situ *hybridization (FISH) mapping located BAC3 on the long arm of platypus chromosome 3 (data not shown). During annotation, no genes were identified with the exception of an intronless class I gene that contained a non-interrupted ORF of 359 amino acids. This gene showed 99% sequence identity to OranPS1-2, a class I sequence previously described as a pseudogene [16]. The class I homology was considered as the reason why the clone had been positive in the BAC colony screen, even though the clone was not MHC-derived.

### Analysis of the platypus core MHC

**Figure 3:**
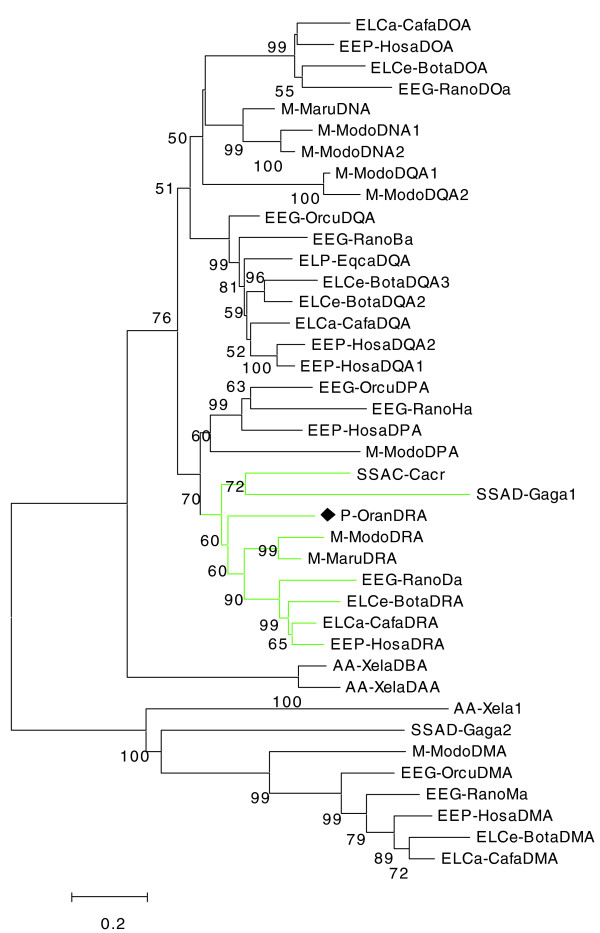
Phylogenetic analysis of class II α chain genes. Cafa, *Canis familiaris *(dog); Hosa, *Homo sapiens *(human); Bota, *Bos taurus *(bovine); Rano, *Rattus norvegicus *(rat); Maru, *Macropus rufogriseus *(red-necked wallaby); Modo, *Monodelphis domestica *(opossum); Orcu, *Oryctolagus cuniculus *(rabbit); Eqca, *Equus caballus *(horse); Cacr, *Caiman crocodilus *(spectacled caiman); Gaga, *Gallus gallus *(chicken); Oran, *Ornithorhynchus anatinus *(platypus); Xela, *Xenopus laevis *(African clawed frog). The abbreviations used to describe taxonomic affiliation of each species in the tree are outlined in Figure 4a. A black diamond highlights platypus DRA. Tree construction using the NJ method is outlined in Materials and methods. Numbers indicate bootstrap support in percent. Branch lengths are proportional to number of substitutions. The scale bar indicates 20% substitutions per site. Sequences used and their accession numbers are listed in Additional data file 10.

In the finished sequence of platypus BAC1 (126,374 bp), seven genes were identified, including two class II genes, two class I genes and three framework genes. One of the two class II genes has a sequence identity of 97% to the platypus class II β chain gene *DZB *[17], and presumably represents a novel *DZB *allele. The other class II locus encodes a class II α chain gene, and phylogenetic analysis suggested orthology with the *DRA *genes of the tammar wallaby (marsupial) and eutheria (Figure 3 and Additional data file 6). The classification of this gene as a platypus *DRA *ortholog is supported by neighbor joining (NJ) and maximum parsimony (MP) analysis. On maximum likelihood (ML) trees, the branching of *DRA *orthologs was not resolved (Additional data file 5). The orthology of the α chain gene to *DRA *genes is surprising, since *DZB *locates to a separate branch in phylogenetic analyses and is non-orthologous to all other β chain subfamilies [17].

The three framework genes were discovered as the platypus orthologs of the immunoproteasome subunit *Psmb8*, as well as *Tap1 *(only partially contained in the BAC sequence) and *Tap2*, which encode for subunits of the antigen peptide transporter. These genes are typically found in the class II region of mammalian MHCs, even though they are involved in the generation and transport of peptides for display by class I molecules. However, in contrast to the MHCs of eutheria and similar to the opossum MHC, platypus possesses an MHC with class II and class I genes being co-localized (Figure 1a,b).

**Figure 4:**
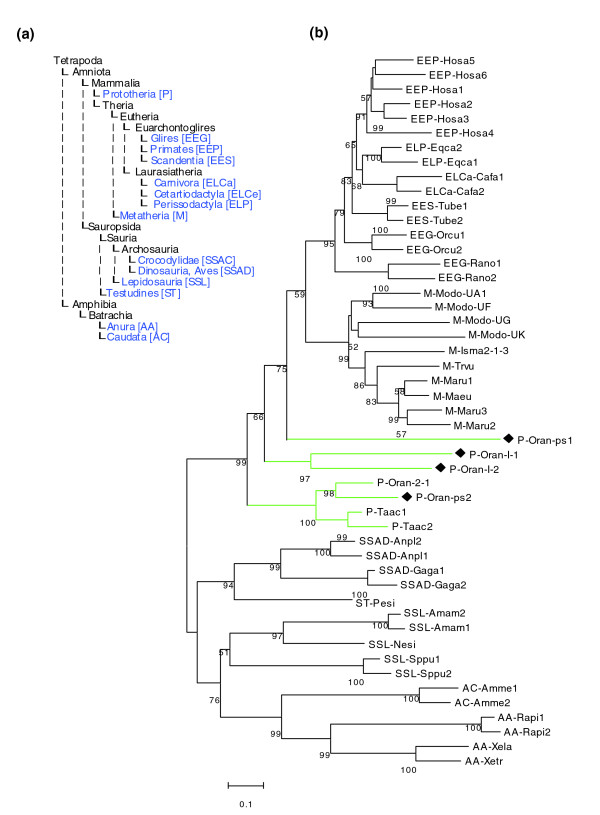
Phylogenetic analysis of platypus MHC class I genes. **(a) **Species tree of taxonomic entities represented in the trees shown in Figures 3 and 4b. **(b) **MHC class I gene phylogenetic tree. Hosa, *Homo sapiens *(human); Eqca, *Equus caballus *(horse); Cafa, *Canis familiaris *(dog); Tube, *Tupaia belangeri *(Northern tree shrew); Orcu, *Oryctolagus cuniculus *(rabbit); Rano, *Rattus norvegicus *(rat); Modo, *Monodelphis domestica *(opossum); Isma, *Isoodon macrourus *(Northern brown bandicoot); Trvu, *Trichosurus vulpecula *(silver-grey brushtail possum); Maru, *Macropus rufogriseus *(red-necked wallaby); Maeu, *Macropus eugenii *(tammar wallaby); Oran, *Ornithorhynchus anatinus *(platypus); Taac, *Tachyglossus aculeatus *(echidna); Anpl, *Anas platyrhynchos *(duck); Gaga, *Gallus gallus *(chicken); Pesi, *Pelodiscus sinensis *(Chinese softshell turtle); Amam, *Ameiva ameiva *(lizard); Nesi, *Nerodia sipedon *(snake); Sppu, *Sphenodon punctatus *(tuatara); Amme, *Ambystoma mexicanum *(axolotl); Rapi, *Rana pipiens *(Northern leopard frog); Xela, *Xenopus laevis *(African clawed frog); Xetr, *Xenopus tropicalis *(Western clawed frog). Black diamonds highlight platypus class I sequences identified in this study. The marsupial Isoodon (Isma2-1-3) is represented by an artificial class I sequence that we generated from three separate overlapping class I sequence entries for this species. Tree construction using the NJ method was as described in Materials and methods. Numbers indicate bootstrap support in percent. Branch lengths are proportional to number of substitutions. The scale bar indicates 10% substitutions per site. Sequences used and their accession numbers are listed in Additional data file 11.

The two class I loci are tentatively named platypus class I-1 and class I-2 genes. We calculated phylogenetic trees including class I genes from eutheria, marsupials, monotremes, reptiles and birds, as well as amphibia. While for eutheria only major lineages were represented in the tree (primates, carnivors, rodents, cetartiodactyls and so on), we included class I sequences from all available marsupial taxons (presently five species), and all monotreme class I sequences currently in GenBank. In addition, the analysis encompassed class I genes from two species of birds (chicken and duck) and four species of reptiles. Amphibian class I genes served as outgroup (Figure 4). All monotreme sequences grouped at the base of the mammalian subclade. Unexpectedly, echidna and platypus class I sequences previously described by Miska and co-workers [16] grouped together, whereas the novel platypus class I-1 and class I-2 genes were in a separate branch (Figure 4; Additional data files 3 and 4). The divergence time between echidna and platypus has been estimated to be approximately 20 Myr [27-29], a similar time span as the reported split between the mouse and rat lineages [30]. It has been shown in the two rodent species that class Ia genes are species-specific, while orthology exists among the more slowly evolving Ib genes [4]. Following this reasoning, the two novel platypus gene classes I-1 and I-2 could be classical class I genes (Ia) and the loci that Miska *et al*. [16] identified in both echidna and platypus correspond to non-classical (Ib) class I genes. However, this hypothesis would need support from expression data.

### Analysis of the platypus MHC *Bat4-Cdsn *interval

The sequence of BAC2 was assembled to a finished length of 194,463 bp. It contained 20 genes in a region that extended from *Bat4 *to *Cdsn *(Figures 1c and 2b). Thus, this sequence covers part of the class III region up to *Bat1*/*Mccd1*, spans a segment that in the human MHC encodes the HLA-B and HLA-C genes, and contains part of the adjacent framework region distal to *Pou5f1*. The gene order and transcriptional orientation of genes is the same as in other mammalian MHCs. However, we could not identify *Ncr3 *in the platypus sequence. *Ncr3 *encodes the natural cytotoxicity triggering receptor 3 and has not been annotated in the opossum MHC but was discovered in both the human HLA and rat RT1. Human and rat NCR3 proteins evolve rapidly and share only 64% amino acid identity (Additional data file 7), which is much lower than the average identity of 88% that has been observed for protein orthologs in these species [31]. Thus, similarity to a putative platypus *Ncr3 *gene may have been too low for detection. Between *Aif1 *and *Lst1 *where *Ncr3 *is located in human and rat, Genscan predicts a platypus gene with correct position and orientation. This prediction could not be verified by homology searches in the NCBI databases, but might be a very diverged version of the *Ncr3 *gene. Alternatively, *Ncr3 *might have been deleted from the platypus genome or might be eutheria specific.

Between platypus proteins and their orthologs in rat or human we found an average of 61-62% amino acid identity (Additional data file 7) and observed much variation in the degree of sequence conservation (range: 27-99%). For instance, BAT1, which is involved in mRNA splicing and transport, was 99% conserved, showing that little innovation in such processes has occurred over the past 160 Myr. In contrast, the framework gene *Lst1*, encoding a protein of unknown function, had an identity of less than 30%.

The region between *Bat1 *and *Pou5f1 *harbors a class I gene block in the MHC of placental mammals. In rat, this region spans 340 kbp and contains 16 class I genes. In platypus, the corresponding region is only 19 kbp in size and contains a class I gene fragment (Oran-ps1) that consists of two exon fragments encoding α2 and α3 domains that are separated by a 283 bp intron. This suggests that the interval once encoded at least one active class I gene in platypus. The sequence identity of the Oran-ps1 translation product to known and new monotreme class I sequences is only 30-40%. We could not find the active counterpart of Oran-ps1 by searching the ENSEMBL platypus whole-genome assembly Oana-5.0 [32], suggesting that Oran-ps1 was inactivated a long time ago. The 283 bp intron within Oran-ps1 is not conserved in other class I loci and cannot, therefore, be used for phylogenetic analysis to date the age of the gene.

### Chromosomal mapping of platypus MHC BAC clones

**Figure 5:**
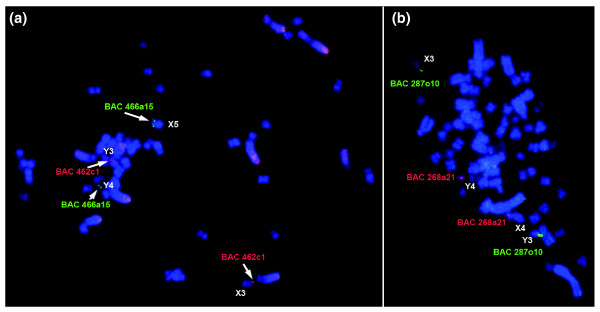
Chromosomal localization of MHC genes in platypus and echidna. **(a) **Platypus BAC clones 466a15 (green) and 462c1 (red) hybridized to male platypus metaphase spreads. Signals were identified on the pseudoautosomal regions of chromosomes Y4X5 and Y3X3. **(b) **FISH mapping of echidna BAC clones 287o10 (green) and clone 268a21 (red) on male echidna metaphase spreads. Signals were detected on the pseudoautosomal regions of Y3X3 and Y4X4.

We determined the localization of the sequenced BAC clones on platypus chromosomes using FISH. Surprisingly, the results showed that the monotreme MHC is not contiguous, and maps to pseudoautosomal regions (PARs) of two pairs of sex chromosomes: BAC1 was localized on X3/Y3 and BAC2 mapped to Y4/X5 (Figure 5a).

### FISH mapping of echidna MHC BACs

**Figure 6:**
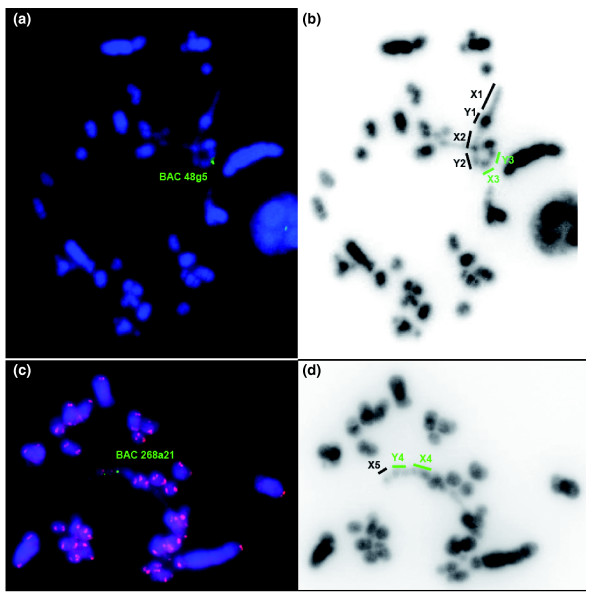
Chromosomal localization of echidna MHC BAC clones in male meiotic metaphase I preparations. **(a) **BAC 48g5 (green) on Y3X3. **(b) **DAPI inverted picture. The elements of the chain are indicated by the bold lines; the elements containing MHC genes are shown in green. **(c) **Hybridization of BAC 268a21 on the meiotic chain. Chromosome telomeres are highlighted by hybridization of a telomere repeat (red). In **(d) **DAPI inverted picture. The elements at the end of the chain are indicated by the bold lines.

To investigate whether the pseudoautosomal location of the MHC is unique to platypus or a common feature in monotremes, we isolated and characterized MHC clones from echidna (see above and Additional data file 1). Two sets of echidna BAC clones were obtained. The first set of BACs covered an interval equivalent to platypus BAC1 and contained the core MHC region with class I and class II genes. The second set of BACs spanned the region encompassing *Bat1 *and *Pou5f1 *(Additional data file 1). The FISH mapping results revealed that the echidna MHC resides, similar to platypus, on two different pairs of sex chromosomes (Figure 5b). The selected BAC containing the core MHC region mapped to X3/Y3, as in the platypus. The echidna BAC that contained *Bat1 *and *Pou5f1 *located to X4/Y4, different to the location in platypus (Y4/X5). In meiotic chains, chromosomes X3, Y3, X4, Y4, and X5 correspond to chain elements 5 to 9. Figure 6 shows the FISH mapping result of two echidna BACs onto echidna male meiotic chains. In the chain, the two echidna BACs map to elements 5, 6, 7, and 8, whereas the corresponding BACs in platypus locate in elements 5, 6, 8 and 9. Thus, platypus Y4X5 occupies a different position in the echidna sex chromosome chain. Since the chromosome morphology of platypus X5 appears to be identical to X4 in echidna, this indicates that platypus X5 has changed its position in the echidna chain.

## Discussion

The platypus MHC is unusual when compared to MHCs from eutherian mammals, because it contains a core region that encodes both class I and class II genes, though such a common class I/II region has also been described in the opossum [10]. The coincidence of compact class I/II regions in both a monotreme and a marsupial supports the hypothesis that this is the ancestral organization of the mammalian MHC. However, a platypus class I gene fragment, Oran-ps1, exists at a position that contains a class I gene block in eutheria. Also, the opossum MHC contains a class I-like MIC gene at this position (Figure 1). Both findings contradict the strict concept of a core MHC and indicate that early mammals already encoded class I genes outside a common class I/II region at positions equivalent to class I gene blocks in eutheria, embedded between framework genes.

The intron-containing class I pseudogene Oran-ps1 and the surrounding region that corresponds to the distal class III region and beyond map to platypus chromosome Y4/X5. In contrast, the core MHC locates to X3/Y3. In the eutherian MHC, class I genes are distributed among several class I gene clusters, separated by hundreds of kilobase-pairs of intervening regions. Despite such distances, concerted evolution takes place between different class I blocks within a species. For instance, the rat RT1-A and RT1-CE regions are more than 1.2 Mbp apart and have a history of transposition events that led to the presence of similar class I genes and shared non-class I gene sequences (gene fragments) in both intervals. Similar but unrelated transposition events were observed within the mouse MHC region, showing that reorganization across large genomic distances is fairly common [4]. These distances seem to be large when considering linear DNA but the regions may be actually juxtaposed in chromatin, facilitating transposition. Oran-ps1 is flanked by *Bat1 *and *Pou5f1 *and these framework genes also flank a class I gene cluster in the eutherian MHC. Thus, Oran-ps1 may have been generated during concerted evolution of class I genes in the platypus MHC at a time when the distal class III region and the core MHC were still on the same chromosome. However, concerted evolution between MHC segments on different platypus chromosomes could be still ongoing, by means of trans-interaction of separate chromosomes [33].

We localized the monotreme MHC to PARs of sex chromosomes. This is the first time MHC genes have been mapped onto sex chromosomes in any mammal or vertebrate. In all mammals investigated so far, the MHC region is located on a single autosome, and in most cases without interruptions. Exceptions are the swine leukocyte antigen (SLA) complex, where the centromere of chromosome 7 separates the class II region from the other parts of the MHC [34] and the recently described class I genes of the tammar wallaby, which were found to be dispersed in the wallaby genome [35].

The multiple sex chromosomes in platypus and echidna pair at the first meiotic division and form a sex chromosome chain during prophase I. PARs are necessary to ensure pairing and segregation of most mammalian sex chromosomes. The ten sex chromosomes of platypus, therefore, would require nine PARs to connect the sex chromosomes at meiosis. The extent of XY homology (that is, the size of PARs; see below) is clearly different among the sex chromosomes. While some PARs are large enough and could easily be identified by chromosome painting, other PARs seem to be much smaller and did not show XY homology using whole chromosome paints [21,22]. We found that MHC genes reside on two of these small PARs in platypus. BAC2 was mapped to the PARs of Y4 and the short arm of X5. This fits with the orientation of X5 in the chain where the short arm lies towards Y4 [21]. Together with the fact that chromosome painting could not detect these PARs, it shows that the Y4X5 shared region is much smaller than the extensive homology found between, for example, X1 and Y1.

PAR sizes vary widely. For instance, the human PAR regions PAR1 and PAR2 differ eight-fold with respect to their sizes, and in the mouse, PAR1 is almost four-fold smaller than in human [36]. The small size of PARs and an obligatory crossover to ensure proper segregation at meiosis results in a high recombination frequency in mammalian PARs [37]. It is therefore interesting to note that the MHC, which is one of the recombination hotspots in the human genome, resides within the PAR, another recombination hotspot.

Another special feature of the monotreme karyotype is the presence of several tiny chromosomes. Initially they where regarded similarly to reptilian microchromosomes [38,39], which was later disputed on the basis of even distribution of chromosome size in the monotreme karyotype [19]. Microchromosomes have long been regarded as heterochromatic elements but have been shown recently to represent the gene rich regions of the chicken genome [40]. The identification of the ten sex chromosomes in platypus revealed that some distinctively small chromosomes are not autosomes, but part of the sex chromosome system (Y3, X4, Y4). The mapping of MHC genes (class I, class II, *Tap1*, *Tap2*) on X3Y3 provides the first evidence for homology to the MHC bearing chicken microchromosome 16. Likewise, five of the MHC framework genes that map on Y4X5 (*Bat2*, *Aif1*, *Tnf*/*Lta*, *Atp6v1g2*) locate to chicken microchromosome 17, establishing another novel synteny link between the genomes of monotremes and birds.

**Figure 7:**
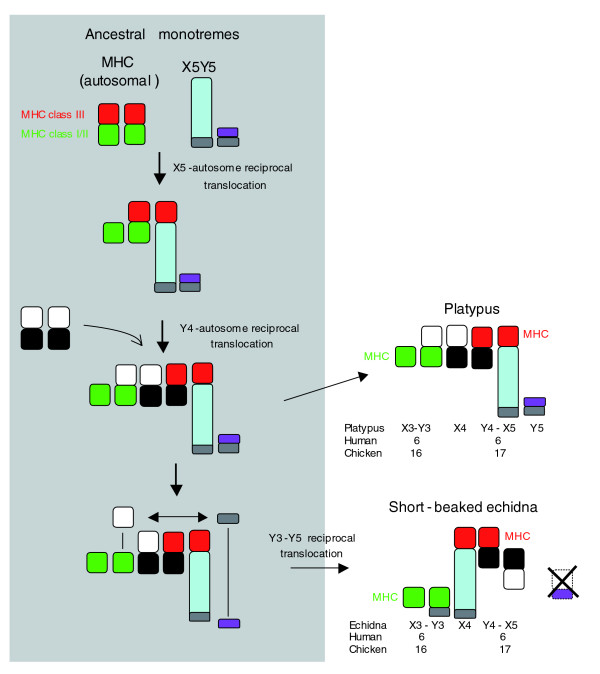
Model for the evolution of elements 6-10 in the male meiotic sex chromosome chains of platypus and echidna. Three events are proposed to have led to the observed arrangement of platypus and echidna sex chromosomes. An initial X-autosome translocation captured the MHC-containing chromosome into the sex chromosome system. In a second step, a sex chromosome-autosome translocation occurred that disrupted the synteny of the MHC. In the echidna lineage, a further translocation between two sex chromosomes took place (see Discussion for details).

Why do MHC-bearing sex chromosomes of platypus and echidnas occupy different positions in male meiotic chains? This work as well as a recent study using chromosome painting and gene mapping has discovered differences in homology and order of the platypus and echidna sex chromosome chains (Rens W., O'Brien P.C.M., Grützner F., Clarke O., Graphodatskaya D., Tsend-Ayush E., Trifonov V., Skelton H., Wallis M., Veyrunes F., Graves J. A. M., Ferguson-Smith M. A., personal communication). Here we present a model that explains the position of MHC BACs in the sex chromosome chins of platypus and echidna. The key feature is a rearrangement between elements of the chain, which explains the different positioning of the MHC bearing sex chromosomes in platypus and echidna as well as the change of orientation of platypus X5 in echidna. We propose that three events led to the observed similarities and differences between platypus and echidna sex chromosome chains (Figure 7). Firstly, an X-autosome translocation captured the MHC-bearing chromosome into the sex chromosome system in a common platypus/echidna ancestor. In a second step, synteny of the MHC was disrupted by recruitment of a pair of autosomes into the chain. In echidna, we propose a further reciprocal translocation between two sex chromosomes, leading to the observed differences in the echidna sex chromosome chain and the positions of the MHC chromosomes contained therein.

The comparative mapping of the MHC in echidna and platypus provides evidence for homology between the sex chromosome systems in platypus and echidna. Our results support the idea that an originally syntenic MHC has been disrupted as a result of the evolution of the platypus and echidna meiotic sex chromosome chain. Following an initial X-autosome translocation in a common platypus/echidna ancestor, subsequent translocations resulted in the differences observed in echidna and platypus sex chromosome systems.

## Materials and methods

### BAC colony arrays, probe design and labeling, and hybridization

Macroarrays and clones were obtained from the CUGI BAC/EST resource centre (Clemson, SC, USA) for the platypus BAC library OA_B and from the AGI BAC/EST resource centre (Tuscon, AZ, USA) for the echidna BAC library TA_Ba. Platypus colony arrays were hybridized with the following oligonucleotide probes: *Bat1*-Gga/Rno, 5'-TACTACGTGAAACTGAAGGACAACGAGAAGAACCGG-3'; *Pou5f1*-Gga/Rno, 5'-CAGACAACCATCTGCCGCTTCGAGGCCCTGCAGCTCA-3'; probe Oan-class I, 5'-AGGACGTCAGCCTGCGGTGCCGGGCCCTCGGCTTC-3', designed from the α3-domain of platypus class I gene Oran2-1 (AY112715); and platypus class II gene probe Oan-class II, 5'-CAGGGAATGGAGCGGCCCATCCTCCCCATGTTGAA-3', designed from the 3' UTR of the *DZB *gene (AY288074).

A *Tap2 *probe was amplified from platypus BAC 462c1 using primers: *Tap2*-Orna-For, 5'-CACCGTGACCCCTCCATCTTCCTCA-3'; and *Tap2*-Orna-Rev, 5'-GTGGGGGAGAGCTCTGGGGGCTGGACG-3'. *Tap2*-positive echidna BAC clones were characterized with the platypus Oan-class I probe (see above) and the two class II gene probes 5'-CGGGAGACATCCAAGTCCAGTGGTTGCGGAATGGA-3' and 5'-TGATGCGTCATGGAGACTGGACCTTCCAGGTCCTG-3'. Pools of three oligonucleotides based on platypus *Bat1 *and *Pou5f1 *were used to identify echidna BACs. Oligonucleotide sequences were: Oran-*Bat1*-1, 5'-GCCTCAACCTGAAGCACATTAAACACTTCATCCTG-3'; Oran-*Bat1*-2, 5'-CGACGTGCAGGATCGCTTCGAGGTCAACATCAGCG-3'; Oran-*Bat1*-3, 5'-CAGGTGGTGATCTTCGTGAAGTCAGTGCAGCGCTG-3'; Oran-*Pou5f1*-1, 5'-ATGGCCGGACACCTGGGTCCCGACTTCGCCTTCTC-3'; Oran-*Pou5f1*-2, 5'-GACCACCATCTGCCGCTTCGAGGCCCAGCAGCTGA-3'; and Oran-*Pou5f1*-3, 5'-CCGCGTCTGGTTCTGTAACCGCCGGCAGAAAGGCA-3'.

We labeled 25 pmol of an oligonucleotide using 25 μCi of γ^32^PdATP (Amersham, Little Chalfont, UK) and 10 u T4-Polynucleotide kinase (New England Biolabs, Ipswich, MA, USA) in a volume of 15 μl at 37°C for 60 minutes. Unincorporated γ^32^PdATP was removed using G-50 MicroColumns (Amersham). Filters were hybridized in Church buffer (0.25 M Na_2_HPO_4_, 1 mM EDTA, 5% SDS) at 58°C over night. Thereafter, filters were washed twice. The first washing step was carried out using 2 × SSC/0.1% SDS wash solution (wash I). The second wash was done using 0.5 × SSC/0.1% SDS (wash II). Each incubation lasted 20 minutes. Filters were exposed on Kodak XAR X-ray film (Sigma-Aldrich, Taufkirchen, Germany) for up to 14 days at -80°C. Procedures for PCR fragment labeling were similar, except that labeling was done with the random primed labeling kit (Roche Diagnostics, Mannheim, Germany), following the manufacturer's instructions, under the addition of 25 μCi of α^32^PdATP (Amersham). Hybridizations and washing steps were carried out at 65°C. The second washing step was done using 0.1 × SSC/0.1% SDS.

### Shotgun library construction and sequencing

BAC clones were grown in 1,000 ml cultures at 37°C with agitation (200 rpm) in a shaking incubator over night. DNA was prepared using the alkaline lysis method and purification on a CsCl gradient in a Beckman centrifuge (VTi65i2 rotor) at 45,000 rpm for 20 h, followed by DNA extraction [41]. Finally, DNA was dissolved in 100 μl 1 × TE. Using a Branson Sonifier 250 (Branson Ultrasonic, Danbury, CT, USA), 10 μg BAC DNA was sheared in a volume of 200 μl to an average size of 1.5 kbp, and after precipitation blunt-ended with Klenow fragment (USB, Cleveland, OH, USA) and T4 DNA polymerase (New England Biolabs). End-repaired fragments were size selected by electrophoresis on a gel of 0.8% SeaPlaque GTG agarose (Biozym, Hess. Oldendorf, Germany) in 1 × TAE buffer (40 mM Tris base, 1 mM EDTA, 20 mM acetic acid). A gel slice that contained fragments of 1.2-2 kbp was cut out, molten at 55°C and incubated at 37°C for 3 h in the presence of 10 u agarase (Sigma-Aldrich) per 100 μl of solution. Following chloroform-phenol extraction, the DNA was precipitated and dissolved in 10 μl 1 × TE buffer. Ligation with 30 ng of dephosphorylated, *Sma*I cut pUC19 and 200-300 ng size-selected DNA was carried out at 16°C overnight, using 400 u T4 DNA ligase (New England Biolabs). A 1 μl ligation was used for transforming 40 μl of ElectroTen Blue *Escherichia coli *cells (Stratagene, La Jolla, CA, USA), following the manufacturer's recommendations. Cells were grown on 22 cm × 22 cm Nunc dishes on LB agar supplemented with 1 ml 2% X-Gal, 250 μl 1 M Isopropyl-β-D-thiogalactopyranosid (IPTG) and 400 μl 50 mg/ml ampicillin per 400 ml medium. Colonies were arrayed into 384-well plates (Genetix, Dornach, Germany) using in-house custom-built robotic devices. Plates contained 2YT medium supplemented with ampicillin (50 μg/ml) and 1 × HMFM freezing mix (0.4 mM MgSO_4 _× 7H_2_O, 1.6 mM Tri-sodium citrate × 2H_2_O, 6.8 mM (NH_4_)_2_SO_4_, 3.6% glycerol, 13.2 mM KH_2_PO_2 _and 26 mM K_2_HPO_4_). Plates were incubated at 37°C for at least 12 h and subsequently shock-frozen on dry ice. Sequencing was carried out on PCR amplified pUC19 inserts, using the BigDye terminator chemistry on ABI3700 automated sequencers. Initial sequence coverage of BACs was up to ten-fold. Thereafter, gaps were closed directionally. Analyzed regions were manually edited in GAP4 [42]. Annotation of assembled BACs was carried out using Genscan and Genewise [25,26]. Exon coordinates in PIPmaker format and predicted translations are available (Additional data files 8 and 9).

### Phylogenetic analysis

We searched the NCBI protein database for MHC class I genes and class II α chain genes in all species groups ranging from amphibians to mammals, based on the NCBI TaxBrowser classification and recorded all the species found. Opossum class I sequences were taken from [43]. We selected only sequences that, by their length, indicated that they were full-coding or nearly so, thereby excluding gene fragments from further processing. The panel of sequences was further reduced by manual inspection of alignments; that is, if several similar sequences representing a gene were found in the data set, only one was kept. We tried to maximize the number of taxonomic entities while keeping the dataset manageable at the same time. In order to achieve this, some taxonomic groups were left represented by a single species only, for example, rat for Glires and human for primates. Also, the number of paralogs per species was reduced in most cases, except for marsupials and monotremes. Multiple alignments were carried out with clustalw [44] using default settings. We calculated NJ trees using Poisson correction as substitution model with homogeneous pattern among lineages, pairwise deletion, and gamma distributed rates among sites with α = 2. We compared the NJ branching to MP and ML trees (Figures 3 and 4; Additional data files 3-6). ML trees were calculated using TreePuzzle [45] and visualized with TreeView [46]. NJ and MP tree calculation and visualization were carried out with MEGA 3.1 [47].

### Preparation of chromosomes and meiotic cells

Mitotic metaphase chromosomes were prepared from established platypus and echidna fibroblast cell lines. Primary cultures were set up from toe web from animals captured at the Upper Barnard River, New South Wales, Australia, during breeding season (AEC permit no. S-49-2006 to FG). The captured animals were killed with an intraperitoneal injection of pentobarbitone sodium (Nembutal, Boehringer Ingelheim, North Ryde, NSW, Austrialia) at a dose of 0.1 mg/g body weight. Meiotic cells and sperm were obtained by crushing the testis. The material was either directly fixed in methanol/acetic acid (3:1) or incubated in 0.075 M KCl at 37°C as hypotonic treatment and then fixed.

### Fluorescence *in situ *hybridization of BAC clones

DNA (1 μg) from BAC clones was directly labeled with spectrum orange or spectrum green (Vysis, Abbot Molecular, Des Plaines, IL, USA) using random primers and Klenow polymerase. Hybridization by FISH to platypus or echidna metaphase chromosomes and echidna meiotic chromosomes was performed under standard conditions. Briefly, the slides were treated with 100 μg/ml RNase A/2 × SSC 37°C for 30 minutes and with 0.01% pepsin in 10 mM HCl at 37°C for 10 minutes. After re-fixing for 10 minutes in 1 × PBS, 50 mM MgCl_2_, 1% formaldehyde, the preparations were dehydrated in an ethanol series. Slides were denatured for 2.5 minutes at 75°C in 70% formamide, 2 × SSC, pH 7.0 and again dehydrated. For hybridization of one half slide, 10 μl of probe DNA was co-precipitated with 10-20 μg of boiled genomic platypus or echidna DNA as competitor, and 50 μg salmon sperm DNA as carrier, and dissolved in 50% formamide, 10% dextran sulfate, 2 × SSC. The hybridization mixture was denatured for 10 minutes at 80°C. Pre-annealing of repetitive DNA sequences was carried out for 30 minutes at 37°C. The slides were hybridized overnight in a moist chamber at 37°C and, thereafter, washed three times for 5 minutes in 50% formamide, 2 × SSC at 42°C and once for 5 minutes in 0.1 × SSC. Chromosomes and cell nuclei were counterstained with 1 μg/ml DAPI in 2 × SSC for 1 minute and mounted in 90% glycerol, 0.1 M Tris-HCl, pH 8.0 and 2.3% DABCO. Images were taken with a Zeiss AxioImager Z.1 epifluorescence microscope equipped with a CCD camera and Zeiss Axiovision software.

### Data availability

The sequence data from this study have been submitted to GenBank under accession numbers EU030442-EU030444.

## Abbreviations

BAC, bacterial artificial chromosome; EST, expressed sequence tag; FISH, fluorescence *in situ *hybridization; HLA, human leukocyte antigen; MHC, major histocompatibility complex; Myr, million years; ML, maximum likelihood; MP, maximum parsimony; NJ, neighbor joining; ORF, open reading frame; PAR, pseudoautosomal region; SLA, swine leukocyte antigen; UTR, untranslated region.

## Authors' contributions

JCD carried out computational analyses, ETA performed FISH hybridizations, RR, FG and HH supervised the work carried out in their labs. HH conceived the study. JCD, FG and HH wrote the manuscript.

## Additional data files

The following additional data are available with the online version of this paper. Additional data file 1 is a figure showing the characterization of echidna BAC clones on Southern blots. Additional data file 2 is a figure showing a phylogenetic tree of MHC class II genes shown in Figure 1. Additional data file 3 is a figure showing an MHC class I gene maximum likelihood phylogenetic tree. Additional data file 4 is a figure showing a MHC class I gene maximum parsimony phylogenetic tree. Additional data file 5 is a figure showing a MHC class II gene maximum likelihood phylogenetic tree. Additional data file 6 is a figure showing a MHC class II gene maximum parsimony phylogenetic tree. Additional data file 7 is a table listing the evolutionary conservation of MHC non-class I, non-class II proteins. Additional data file 8 is a table listing gene models in PIPMaker format and ORF translation for platypus BAC 462c1. Additional data file 9 is a table listing gene models in PIPMaker format and ORF translation for platypus BAC 466a15. Additional data file 10 contains Additional data file 10 is a table listing accession numbers for MHC class II genes used for phylogenetic analysis in Figure 3. Additional data file 11 is a table listing accession numbers for MHC class I genes used for phylogenetic analysis in Figure 4.

## Supplementary Material

Additional data file 1 Characterization of echidna BAC clones on Southern blots. Click here for file

Additional data file 2 Phylogenetic tree of MHC class II genes shown in Figure 1. Click here for file

Additional data file 3 MHC class I gen? maximum likelihood phylogenetic tree. Click here for file

Additional data file 4 MHC class I gene maximum parsimony phylogenetic tree. Click here for file

Additional data file 5 MHC class II gene maximum likelihood phylogenetic tree. Click here for file

Additional data file 6 MHC class II gene maximum parsimony phylogenetic tree. Click here for file

Additional data file 7 Evolutionary conservation of MHC non-class I, non-class II proteins. Click here for file

Additional data file 8 Gene models in PIPMaker format and ORF translation for platypus BAC 462c1. Click here for file

Additional data file 9 Gene models in PIPMaker format and ORF translation for platypus BAC 466a15. Click here for file

Additional data file 10 Accession numbers for MHC class II genes used for phylogenetic analysis in Figure 3. Click here for file

Additional data file 11 Accession numbers for MHC class I genes used for phylogenetic analysis in Figure 4. Click here for file
