# Supplementary material for: Disruption and pseudoautosomal localization of the major histocompatibility complex in monotremes
Source: Genome Biol. 2007 Aug 29;8(8):R175. doi: 10.1186/gb-2007-8-8-r175 (PMC2375005; doi:10.1186/gb-2007-8-8-r175)
Supplement: Additional data file 1 — Characterization of echidna BAC clones on Southern blots. [file gb-2007-8-8-r175-S1.doc]

SUPPLEMENTARY FIGURES


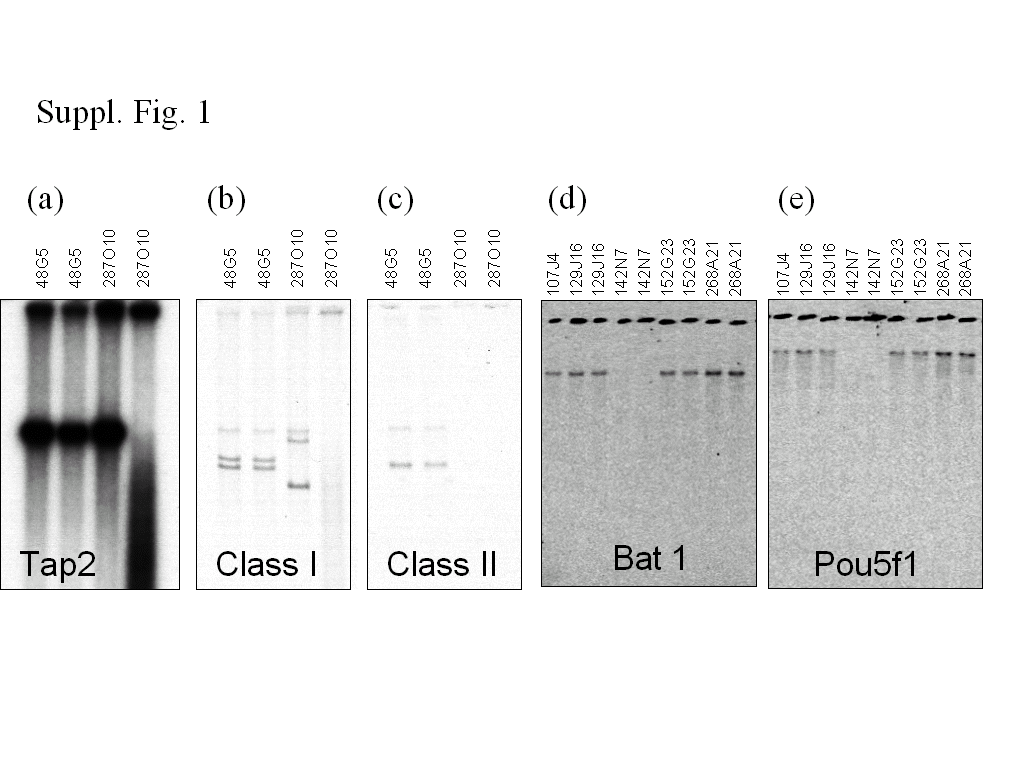


Suppl. Fig.1. Characterization of echidna BAC clones on Southern blots. (a-c) Clones identified with a *Tap2* probe. Clone 48g5 contains class I and class II sequences, clone 287o10 is positive for class I, but class II genes can not be detected. (d, e) Confirmation of *Bat1*/*Pou5f1* positive clones from colony screen. All clones except 142n17 are true positives.
