# Supplementary material for: Disruption and pseudoautosomal localization of the major histocompatibility complex in monotremes
Source: Genome Biol. 2007 Aug 29;8(8):R175. doi: 10.1186/gb-2007-8-8-r175 (PMC2375005; doi:10.1186/gb-2007-8-8-r175)
Supplement: Additional data file 2 — Phylogenetic tree of MHC class II genes shown in Figure 1. [file gb-2007-8-8-r175-S2.doc]

SUPPL. FIG. 2

Suppl. Fig. 2. Phylogenetic tree of class II genes present on Fig.1, panel b, for orthology relationship determination. Hs, *Homo sapiens* (human); Rn, *Rattus norvegicus* (rat); Md, *Monodelphis* *domestica* (opossum); Oa, *Ornithorhynchus anatinus* (platypus).
