# Supplementary material for: Disruption and pseudoautosomal localization of the major histocompatibility complex in monotremes
Source: Genome Biol. 2007 Aug 29;8(8):R175. doi: 10.1186/gb-2007-8-8-r175 (PMC2375005; doi:10.1186/gb-2007-8-8-r175)
Supplement: Additional data file 4 — MHC class I gene maximum parsimony phylogenetic tree. [file gb-2007-8-8-r175-S4.doc]

SUPPL. FIG. 4: Class I, Maximum Parsimony tree

Search Options : CNI (level=1) with initial tree byRandom addition (100 reps)

Gaps/Missing Data : Use all sites
