# Supplementary material for: Disruption and pseudoautosomal localization of the major histocompatibility complex in monotremes
Source: Genome Biol. 2007 Aug 29;8(8):R175. doi: 10.1186/gb-2007-8-8-r175 (PMC2375005; doi:10.1186/gb-2007-8-8-r175)
Supplement: Additional data file 5 — MHC class II gene maximum likelihood phylogenetic tree. [file gb-2007-8-8-r175-S5.doc]

SUPPL. FIG. 5

Class II, Maximum Likelihood tree, outgroup HosaDMB (GenBank AAB60387)

TreePuzzle Prameters:

v Approximate quartet likelihood? Yes

n Number of puzzling steps? 1000

o Display as outgroup? O-HosaMICB (19)

e Parameter estimates? Exact (slow)

x Parameter estimation uses? Neighbor-joining tree

m Model of substitution? Auto: JTT (Jones et al. 1992)

w Model of rate heterogeneity? Gamma distributed rates

a Gamma distribution parameter alpha? Estimate from data set

c Number of Gamma rate categories? 8
