# Supplementary material for: Disruption and pseudoautosomal localization of the major histocompatibility complex in monotremes
Source: Genome Biol. 2007 Aug 29;8(8):R175. doi: 10.1186/gb-2007-8-8-r175 (PMC2375005; doi:10.1186/gb-2007-8-8-r175)
Supplement: Additional data file 7 — Evolutionary conservation of MHC non-class I, non-class II proteins. [file gb-2007-8-8-r175-S7.doc]

Suppl. Table 1

| **1** | **2** | **3** | **4** |
| --- | --- | --- | --- |
|  | Platypus vs. Rat | Platypus vs. Human | Rat vs. Human |
| *Psmb8* | 194/274 (70%) | 196/274 (71%) | 245/274 (89%) |
| *Tap2* | 452/706 (64%) | 463/705 (65%) | 530/701 (75%) |
| *G4* | 140/308 (45%) | 133/311 (42%) | 221/294 (75%) |
| *Apom* | 108/189 (57%) | 106/188 (56%) | 155/189 (82%) |
| *Bat3* | 828/1102 (75%) | 842/1140 (73%) | 1010/1153 (87%) |
| *Bat2* | 1652/2243 (73%) | 1650/2238 (73%) | 1934/2169 (89%) |
| *Aif1* | 104/139 (74%) | 108/139 (77%) | 132/147 (89%) |
| *Ncr3* | n.a. | n.a. | 115/179 (64%) |
| *Lst1* | 37/135 (27%) | 31/105 (29%) | 22/35 (62%) |
| *Ltb* | 140/313 (44%) | 123/252 (48%) | 185/310 (59%) |
| *Tnf* | 134/239 (56%) | 136/237 (57%) | 185/236 (78%) |
| *Lta* | 106/181 (58%) | 103/181 (56%) | 149/205 (72%) |
| *Nfkbil1* | 223/359 (62%) | 221/359 (61%) | 351/381 (92%) |
| *Atp6v1g2* | 103/116 (88%) | 104/118 (88%) | 114/117 (97%) |
| *Bat1* | 422/425 (99%) | 424/425 (99%) | 426/428 (99%) |
| *MCCD1* | n.a. | 63/114 (55%) | n.a |
| *Pou5f1* | 212/370 (57%) | 222/370 (60%) | 304/360 (84%) |
| *Tcf19* | 173/267 (64%) | 237/345 (68%) | 215/266 (80%) |
| *Hcr* | 314/670 (46%) | 354/683 (51%) | 552/758 (72%) |
| *Spr1/Psors1c2* | 89/135 (65%) | 98/135 (72%) | 99/136 (72%) |
| *Cdsn* | 257/589 (43%) | 283/584 (48%) | 373/553 (67%) |

Web supplement Table 1. Evolutionary conservation of non-class I, non-class II proteins encoded within platypus BACs 466a15 and 462c1. Comparison to their rat and human orthologs. Genes *Bat4* and *Tap1* were only partially encoded within the BACs and were not included in the analysis. Rat protein sequences were taken from BX511170 [4], except for *Psors1c2* and *Cdsn* for which the rat annotation was refined using Genewise [26]. Human protein sequences were from RefSeq, selecting the longest available isoform of the protein. Comparison was carried out by pairwise BLAST ([www.ncbi.nlm.nih.gov/blast/bl2seq/](http://www.ncbi.nlm.nih.gov/blast/bl2seq/)).

Column 1, gene name; Column 2, comparison of platypus and rat protein; Column 3, comparison of platypus and human protein; Column 4, comparison of rat and human protein.

n.a.: not applicable. One of the species lacks the respective gene.
