# Supplementary material for: Disruption and pseudoautosomal localization of the major histocompatibility complex in monotremes
Source: Genome Biol. 2007 Aug 29;8(8):R175. doi: 10.1186/gb-2007-8-8-r175 (PMC2375005; doi:10.1186/gb-2007-8-8-r175)
Supplement: Additional data file 8 — Gene models in PIPMaker format and ORF translation for platypus BAC 462c1. [file gb-2007-8-8-r175-S8.doc]

Suppl. Table 2

Platypus BAC 462c1 (BAC1)

< 852 16048 Class II - DZB

852 1197

2021 2044

3107 3217

3747 4028

10270 10539

15895 16048

> 17591 23110 Class II - DRA

17591 17672

21732 21977

22485 22766

22959 23110

> 59960 66946 Class I

59960 60029

61076 61342

63175 63459

64886 65152

66214 66303

66906 66946

< 80309 91726 Class I

80309 80349

81605 81712

81911 82192

82332 82610

83880 84146

91663 91726

< 95871 107574 Tap2

95871 96059

97057 97193

98404 98563

98919 99092

99355 99543

100259 100387

100966 101163

101992 102197

103403 103533

105793 105907

107055 107574

< 111245 119295 Psmb8

111245 111333

112125 112329

113200 113329

114781 114892

116907 117054

119167 119295

< 125391 125606 Tap1

125391 125606

Peptide sequences of gene models in platypus BAC 462c1 (BAC1)

>Class II - DZB

MASLLVPWSLWAAAVAVALMVLTSPGAGGRETPEHFLYQTKS

ECYFYNGTERVSFVDRYMYDRQEIVRFDSDVGVFVAVTELGRPDAQYWNSLQDFLDEKRT

EVDRVCRHNYGVGEQFTVQRRVQPTVKVSQTNAKPLEQHQTLTCSVTGFYPGDIQVQWLR

NGQEQKDGVVHTDLMRHGDWTFQVLVILEMTPRSGDVYVCQVEHGSLQKPITVEWRAQSE

SARSKMLSGVGGLVLGLIFLAVGLTVHFKGRKGHTVSQPAGLLS*

>Class II - DRA

MAPTTALALRVLSLASLLATHCTRAVKWEHRIIQAEFSQTHSPTGEFMFEFDGDEIFHVD

LDRKETVWRLADFSNYASFEAQGGLANLAVDKANMDILIKRSNHTPATNVAPEVTVFPEN

PVEMGQPNILICFVDKFSPPVVNISWLRNGQPWSKGASETDFYPRTDHSFRKFHYLPFIP

SANDFYDCKVEHWGLEEPLLRHWEPKVPSPLTETKETLVCALGLAVGLVGIIVGTILIVR

GLRSGTAPRPQGPL*

>Class I-1

MGTLAFSLLLFLEVPQLPGSRAAGSHFLRYFFTGVSRPGPGLPAFTAVAYVDDQQLGHFD

SDGGMSIPSAWWILETDVGDHLKDLKRIATGLARDSQDSLRDLPRYYNQTEGGSHSIQTM

FGCEVEDNGTVRNGYYQVGYDGQDYIALERNHKNMTWVAADTVAQITKNMWEENEKFISR

LRNHLECDCPKWMKIYLRLGGDSLNRKRLTHHPSPDGDDVTLRCRALGFYPSRIRMRWQR

DGEDLTRDTEQVETRPGGDGTFQTWTAAVGVPRGQERRYTCIVQHDGLAEPLIVRWEPSP

PISGFVAIILILTAVILGVVLWRKRLGGKDGNDNSTDSE*

>Class I-2

MPLQKDLVLIPTLPLVCSVSLGSHFLRYFYTAVSRPGPGVPAFTAVGYLDDQQFVRFDSI

RQKAEGLTTWIQGGQGPDYWEQQNQDLRGTQQIFLQNLQVALSYYNQSEGGYHSYQEMYG

CELRTDGSTGKAYDRYGYDGQDYITLDLDTLTWTAANPEAQYTKRKWEANKKKLELEKAY

LQGQCVYWISEYLKLGGDSLNRTEPPSVRVTRHPSQDEDNVTLRCQALGFYPADIRMRWQ

RDGEDLTRDTEHVETRPGGDGTFQKWTAVVGVSHGQEQRYVCVVDHDGLANPLAVGWVSD

LSPRKAVIMGALATVLIVTAVLAGVVILRKRRPGEQQSYYIPAASE*

>Tap2

MPPRILLLCGPLVLLDLTLLWLLKVTPWALLPLSLPRLWIEGVLRLGALWGALGTLQGSL

GPKAGGGLEGLLPSLCLSPPLFLSLRALVARTSDAPPSLMASSSWSWLLLSYGAVALSWA

LWAVLSPPKAPASTQEKERGRELRVMVRRLLDLSRPDLPWLVGAFLFLTAAVIGETTIPY

YTGRVIDILGGSFEPEAFVSTIGFMCLFSLGSSVSAGCRGSLFMVAMSRLNLRLRQILFS

SLLRQDLEFFQETKTGELNSRISSDTSLMSRWLPLNANVFLRSLIKALGLYGFMVPLSPR

LTLLSLLDLPLTLTAEKVYNSRNRAVLQAIQEAVAAAGQVVREAVGAMETVRSLGAEQEE

ARRYEAALERLRLLHWRRDLERALYLLFRRALQLGMQVLMLWCGLKQILAGDLTRGGLIS

FLLYQENVGTYVKTLVYICGDMLSNVAAAEKVFGYLDREPRRPPPGKLAPETLRGHVEFQ

DVTFAYSGRPNDTVLKGVTFTLRPGEVTALVGPNGAGKSTVAALLQGLYQPTAGRLLLDG

QPLDRYRHRYLHAQVSVVAQEPVLFSGSVRDNITYGLESCREEEVRAAVRDACADGFVAD

LAQGLDTDVGEKGSQIATGEKQRLAIARALVRHPKVLILDEATSALDTECEKAMQKSVLG

GGRTVLVIAHRLGTVQKADQILVLEEGRVVERGTHEQLLELRGLYHRLVQRELTD*

>Psmb8

MALQQVCGPSPGWKDCLSSPPSLSGRSPFSSGTLDFEVPPGLQPSAFLKSLVGNRDDRVQ

IKLFKGTTTLAFNFQHGVVVAVDSRATAGNYICSQMVNKVIEINPQLLGTMSGSAADCQY

WERLLAKECRLYHLRNGERISVSAASKLLSNMMCQYRGMGLSMGSMICGWDKKGPGLYYV

DEDGVRLSGLMFSTGSGRNYAYGVMDSGYRYDLSPEEAYDLGRRAIVHATHRDGYSGGMV

NMYHMKQDGWIKVEQTDVNDLLLSTLEARV*

>Tap1

VERLLYDHGERTSRTVLLITQSLSLAERAAQILFLEEGVLREVGTHEELMSRGGRYWDMV

AAQWASAEPED*
