# Supplementary material for: Disruption and pseudoautosomal localization of the major histocompatibility complex in monotremes
Source: Genome Biol. 2007 Aug 29;8(8):R175. doi: 10.1186/gb-2007-8-8-r175 (PMC2375005; doi:10.1186/gb-2007-8-8-r175)
Supplement: Additional data file 9 — Gene models in PIPMaker format and ORF translation for platypus BAC 466a15. [file gb-2007-8-8-r175-S9.doc]

Suppl. Table 3

Platypus BAC 466a15 (BAC2)

> 837 1290 Bat4

837 1290

> 5429 6151 G4

5429 6151

< 7273 11002 Apom

7273 7298

7549 7644

8850 8948

9337 9410

10413 10567

10889 11002

> 13933 25133 Bat3

13933 14061

16859 16976

17160 17359

17497 17550

17696 17770

17983 18224

18479 18608

19289 19486

19950 20187

20448 20519

20830 20945

21056 21140

21393 21700

22151 22439

22767 22936

23029 23082

23380 23482

23599 23723

23906 24052

24152 24211

24304 24453

24548 24655

25038 25133

< 25519 37428 Bat2

25519 25662

25768 25858

25949 26037

26259 26483

26638 26736

26842 27052

27158 27240

27369 27560

27704 27778

27938 28069

28166 28399

28498 28580

28689 28818

29000 29121

29228 29481

29583 31448

31605 31815

31938 32238

32378 32568

32831 33353

33536 33749

34228 34318

34630 34772

34868 34947

35085 35218

35726 35869

36018 36090

36179 36278

36704 36881

37317 37428

< 41950 44960 Aif1

41950 42034

43181 43343

44184 44225

44361 44427

44590 44651

44951 44960

< 57294 59686 Lst1

57294 57476

57848 57981

59363 59479

59641 59686

> 69705 74889 Ltb

69705 69839

72626 72770

74444 74889

< 82734 84720 Tnf

82734 83149

83768 83812

84489 84720

< 88728 89511 Lta

88728 89146

89403 89511

< 101855 103542 Nfkbil1

101855 102405

102629 102853

103266 103542

> 106470 108142 Atp6v1g2

106470 106551

106774 106874

107969 108142

> 114373 119739 Bat1

114373 114583

114670 114797

115030 115122

115923 116106

116223 116341

118774 118905

119009 119118

119214 119358

119506 119739

< 120467 121035 MCCD1

120467 120691

120898 121035

> 128809 129552 class I pseudogene containing STOPs

128809 128992

129245 129552

> 140358 144833 Pou5f1

140358 140739

142707 142847

142947 143077

144302 144463

144573 144833

< 146047 148616 Tcf19

146047 146257

147258 147807

148373 148616

> 150959 159353 Hcr

150959 151019

151447 151630

152378 152639

153598 153761

153869 154004

154233 154343

154640 154789

155025 155135

155953 156059

156875 156987

157148 157196

157330 157466

158251 158439

158849 158950

159064 159353

> 161704 162657 Spr1

161704 161755

162308 162657

> 177587 181997 Cdsn

177587 177671

180304 180733

180860 181997

Peptide sequences of gene models in platypus BAC 466a15 (BAC2)

>Bat4

SPSPEPRYCDVCQTCFRDANHASSTAHLLALPRGPRPPHPPPGFPVSSPGFRLLLRGG

WEPGTGLGPHGQGRAEPVATVLKRDQEGLGYGQPPRPRVTHFPAGDPRAVRGPDRDLRTP

RAATLGKRKEKRREEKSRAWERNLRTYMNLDF*

>G4

MFLRRLAGWLPRPWGRQKAGDPPGPAPGPGPGLPRPESSPENSGSEWDSAPETAGDGEDP

GPPGPPRARDGLGVPPVQPGRRQRLLGWIRSRRTGELGGGPQFSGSLSPAERLRICPNLT

RHLLDLLLSALLALSSPPLRVLLEALGFRGPAGLWLHGLLSFVLALHALHALLALLTAYP

VHFACLFGLLQGLVLAVSLREEEEEEEAASAPREAGDGSREAWPDGEDEEEEDVEGTPAL

*

>Apom

MIHQFWTYLLYLYWSLQNSISRCPGPTPLVIPGPHGEEFPRTYLGYWHFIAGSAPTPDPL

ATFDPVDNILFHLVPGSTPLELYLRASIRMKNGLCVPREWTYLLAKGTTNLRIEGRPNMK

TELFSAPCAESIILKETGQDYERFLMYSRSPAPPARCVNDFQALVSCWNFTLLQTPRQQD

ACMLTSS*

>Bat3

MDPGGGGGGGGPGPGPDMEEPADLEVSVKTLDSQTRTFTVGAEMTVKEFKEHIAAAVSIP

PDKQRLIYQGRVLQDDK

KLQEYNVGGKVIHLVERAPPQTQGPSSGGASRAGSPSAPHAGAPPAGPRGPGAPVHDRNA

NSYVMVGTFNLPSDGSAVDVHINMEQAPIQSEPRVRLVMAQHMLRDIQALLARLEPQPGQ

QGQQGQQQGQALLAESGPPRPGPPGQTDGETSPREPTETREPTETREPEDGVAARGTGPA

PGPAPAPAPEGNAAPNHPSPAEYAEVLQELQRVESRLQPFLQRYRAILGAAATTDYNNNT

EGREEDQRVINLVGESLRLLGNTFVALSDLRCNLSATAPRHLHVVRPMSHYTAPMVLQQA

AIPIQINVGTTVTMTGSGARPGPTDTTPTSGQTSSPTPSPTSGEPGPDGAPSGPAPPQAA

GPPRLIRISHQSVEPVVMMHMNIPDSGSQTGGTSSASTASTGLPGQGLGQQVSGFPAAPT

RVVIARPTPPQARPPHPGGPPPAPGATIPVPGSNASLAQMVSGLVGQLLMQPVLVAQGAS

GLGAPQAPATASASAGTTNTATTAGPAPGGPAQPPPPPPPGPPQAEVQFSQLLGSLLGPG

VPGGPGTAGGATSVGSPTITVAMPGVPAFLQGMTDFLQATQTAPPPPPPPPPPPAPEQAP

AAAPPGSPPAGPGGAGGGPEALPPEFFTSVVQGVLSSLLGSLGARAGSGESIAGFIQRLS

GSSNIFEPGADGALGFFGALLSVICQNLSMVDVVMLLHGHSQPLQRLQPQLRGFFHQHYL

GGREPTGPAIRRATHTLITGLEEYVRDSFASVQVQPGVDITRTNLDFLQEQFNGIAAHVL

HCTDSSFGVRLLELCNQGLFECLALNLHCLGGQQSALTNVINGRIRRLSGGVNPSLVSWL

TTMMGLRLQVVLEHMPVGPDQVLRYVRRLGEPPQPPPEEPMDVQGAERAPPEPERENASP

APGTTAEEAMSRGPPPAPEGPPPLEEQDGAAAAESEPWAAAVPPEWVPIIRQDLQTQRKV

KPQPPLSDAYLSGMPAKRRKLRSDLQQRLRADPNYSPQHFPNAQRAFMDEP*

>Bat2

MSDRSGPTAKGKDGKKYSSLNLFDTYKGKSLEIQKPAVAPRHGLQSLGKVAIARRMPPPA

NLPSLKAENKGNDPNVSLVPKDGTGWASKQEQTDPKSSDASTAQPPESQPPPASQTPASS

QPKRPPAGPENAPSAPSGAKSWAQASVTHGAHGDGGRASSLLSRFSREEFPTLQAAGDQD

KAAKERESAEPSSGPGPSLRPQNSTTWRDGGGRGPEELEGPDSRLHAGLQPAGPPQFPPY

RGMMPPFMYPPYLPFPPPYGPQGPYRYPTPDGPSRFPRLAGPRGAGPQLRLAEPVSRPAI

LKEDNLKEFDQLDQENDDGWAGAHEEVDYTEKLKFSDEEDGRDSDEEGAEGRKEPRPAGE

ERPPGSEADGPKGGSPPAEPPPPKTAWAENSRPPEGEPGPPPPKPPPPHRGPAGNWGPPG

DYPDRGGPPCKPPAPEDEDEAWRQRRKQSSSEISLAVERARRRREEEERRMQEERRAACA

EKLKRLDEKFGAPDKRLKAEPPAPAVAATPPAPVLVPAPAPAAVTTAAPVPAVAPLPPGP

PAALPAPSTAPEKEPREPGVALAVTPAPAPAAAPPPAPAAVPAPAGGDGTGGFDPGPAEP

PLPTKDGPELLDEAPQPATPPTPKAEPKADGPGPARPPPGQGLGYPKYQKSLPPRFQRQQ

QEQLLKQQQQQQWQQQQQQNSAPPTPVPPSPPQPVALGPVPAPPAPPQPPKALYPGSLGR

PPPMPPLNFDPRWMMIPPYMDPRLIQGRAPLDFYPPGVHPSGLVPRERSDSGGSSSEPFD

RHPTPLLRERGTPPVDAKLAWAGDVFATGNADPRPLPSPLRQTADEEDKGMRSETPPAPP

PPPYLATYPGFPENGAPGPPLSRFPLEEPAPPGTRPGPWAPADETPKLPAPPKKEPPKEE

PPPGGPEAGRKPPRGANGGPPLRRESRTETRWGPRPGSSRRGPPEEAGVTGAPPPPRRAG

PIKKPLGLPKAEEPPAKAPDTAEETPRPDPLKPPKVKAGMVAAMGKEPPSGGGGPLSPSP

RPRRDYSSEGPGPCRGRGRGEYFARGRGFRGTYGGRGRGGRGGREFRSSREFRAEDGRGV

GGPGPHPLPPRGRTASETRSEGSEYEEVPKRRRQRGSETGSETHESDLAPSDKEVAVPPA

APPPLPPHPSPAPGARFPAARGGRVFTPRGVPSRRGRGGGRPPVMAGWSPPAKALGPKKP

LGAPPPPPSKEAVVVIVGGGGGAKEKAPGGGGGPIPGPPPRGGLEDGERPRRRRHGRAQQ

QDKPPRFRRLKQERDNAARGADGPSPAPAPVPAPAPDEPPDAGPGPAPAPPAPPPAPASS

QPAQPPPRRAAAKSPDLSNQNSDQANEEWETASESSDFAGERRGDKEAPPAALLTPKAGG

GGPPGGPGPRPEPSHRARDLSKRSFSSQRPGMERQNRRPGPPGKAGGGAGGGPGGRAGPS

RGDKRNWPSPKNRSRPPEEQPVGLPLPPPPPSSSAVFRLDRVIHSDPAGIQQALAQLSSR

QAAPGSVPPRPKPGPPAAPRAPAHYEPPRASSHGAGGTDPHLVEPGPAARGVDSAGASPF

PPKRRERGPPRKPELLPPEEAPPPPHGSAFPGPKPEGPGPRGEPRDPGTETLAPHIWNRL

HSTASRKSYRPGSEPWMEPLTPLEDGPGPEMSQSDSGVDLSGSSQVSSGPCSQRSSPDGG

LKAVGEGAPKRPGGPSPLSATSAEGPPGPEPPDPSRRRPPAPRDGDKKEPPPPPGPIGTE

RGQRADRGQESAGAPRDPHRPGPPIQFGASDKDSDLRLSLAEGVKPEEDLTPSGGEGAAP

GPRDWELLPPRSQPAEPRPKSLGPPHCGPEPGPSPGPSPGPSGPRLYPELFYGSTGPPGS

QVPGAAVDSTLHPNTGAFRPSTPSLHPFRSQPLYLPPGPAPGSALLSGVALKGQFLELSA

LQAAELGKLPGGGVLYPPPSFLYPPAFCPSPLPDPALLQVRQDLPSPSDFYSAPLQPGGQ

NGFLPPAAPAQQMLLPVVESQLPVVNFSSLAPAPPPAPPPPPPPLSLLPVGPALQPPSLA

VRPPPVPPARVLPSSARPFPPSLGRAELHPVELKPFQDYRKMGNLGGAGARTPTAARPFS

GLGSRLKAPPNGYSGIFRTQRFDLYQQASPPDALRWTPKPWDRGGPPPREGPPRRPEETQ

PRPGDKDPGLPPPR*

>Aif1

MGLGGKAYGEFLLQQEQRLDGINQQFLDDPKYSTDEELPQKLRAFSKKYMEFDLNGDGDIDIM

SLKRMLEKLGTPKTHLELKKMIGEVAGGQRETISYQDFVRMMLGKKSAIFKIILMYEEKG

REQEKPTGPPAKKTISELP*

>Lst1

MIFYLPQRYEQSLTNSNTSLDKICCFLIGLAGVTIGLVVAVIILAICLCRLQKRGAELQA

LWDRESKATGSHSDHYSSLQFSQGLGEASCSGCFALSLRAQDNSLELREEQELHYASLRR

LPIGDTGEGAKEEGPKEETRNPTEDFSSDYASVVKRPDPS*

>Ltb

MGARGGEPGRPLLAVVGAAVLGTLLVCVPLTALVTMALGPRSWGQEAAPEPPGGQRQQQM

GEDPSRDPGVLPPGPAEDWGLEETLRNPAAHLIGIQGHGPGHLLHWVTGHEEAFLKSGAR

LLGTARLALPREGVYYLYCHVGFRGRGSGGHPGAGITVSSRLYRVGGGYGAGEVELMLQG

AETVTPPPGDGRGPGGLWHTSVGFGGLARLGGEERIFVNVSHPNMVDYRRGKTYFGAVRV

G*

>Tnf

MSTESMLRDIELAGEPAGKTAGAQRPGHCLCLSLVSFLLVAGATTLFCLLHFGVIGPQDR

EVRELSQIHTCTHTHTYQSYQPSAEKPAAHVVANSQEDKKLVWVGGRANALLEGNVILDK

NQLVVPASGLYLVYSQILFKDSSCPANPDDSPILTHNVSRFSDSYEYEVSILSAIKTPCQ

GGAKGTWYEPIYQGGIFRLNQGDRLSSQTNSPEYLDFSMEGQVYFGIIAL*

>Lta

XGHLGGPGVPRSPHLTAHLDLRRRLLRDAPKPAAHLIGDPSRPGSLLWRDNSDHA

FLHHGFRLQNNTLLVPSSGVYFVYTQTVFSGPGCTEDGQGTLAPNYLSHEVLLFSTQYPI

HVPLLSAQKSVCPGPKGPWIKSIYQGAVFSLMAGDQLSTRTEGVSNLLLVPGSVFFGAFA

V*

>Nfkbil1

XSKSAMGSSSRRHRRERRFRHYLSTGHLGRARALLRRHPELDVDSGQPPPLHRACARRDPPALRL

LLRRGADPTRQDRHGDTALHAAARQGPDAYRDLFLPLLSRCPAAMGMRNEWGETPGELLG

WGAPPEPPPPQEEPSREAEDAEWRWKLYEELQDEWQEILGRFEDDDPQEEPETYSAWTER

LAREYARRRRQREEPRRPRPPPASTPHGRGWPDEGEEQRLYRERAWAKELELQESRARRE

REKEQERAEERAGDPGRRLWGFKDVPWPSPGGGDAEAMAATLLAGGPPAEPAEPFRKFLR

AQRVLWHPDRFLQRFGSRLDPRERARVLEAVTALSQALNRRAEGLK*

>Atp6v1g2

MASQSQGIQQLLQAEKRAAEKVADARKRKARRLKQAKEEAQMEVEHYRREREQEFQTKQQ

AAMGSQGNVSAEVEQGTRRQVQDMQSAQQRSRERVLARLLALVCDVRPQIHLNYRIAA*

>Bat1

MAENDVDNELLDYEDDEVETAAGGDGAEAPAKKDVKGSYVSIHSSGFRDFLLKPELLRAI

VDCGFEHPSEVQHECIPQAILGMDVLCQAKSGMGKTAVFVLATLQQLEPVTGQVSVLVMC

HTRELAFQISKEYERFSKYMPNVKVAVFFGGLSIKKDEEVLKKNCPHIVVGTPGRILALA

RNKSLNLKHIKHFILDECDKMLEQLDMRRDVQEIFRMTPHEKQVMMFSATLSKEIRPVCR

KFMQDPMEIFVDDETKLTLHGLQQYYVKLKDNEKNRKLFDLLDVLEFNQVVIFVKSVQRC

IALAQLLVEQNFPAIAIHRGMPQEERLSRYQQFKDFQRRILVATNLFGRGMDIERVNIAF

NYDMPEDSDTYLHRVARAGRFGTKGLAITFVSDENDAKILNDVQDRFEVNISELPDEIDI

SSYSESHPPVPCVPCPQPQPTSPPPPPAAIR*

>MCCD1

MGPPLPWLSRCYRLLPRALPTKGLGFSGNNRLPPEDAHAGETHSTERRRQQEERQCGFPG

DRRAELAQVEQLLEQQLELYQALLEGQDGAWEAQALVLKVQKLKEQMRRHREGPGERDAI

*

>ClassI-pseudogene-containing-STOPs

GSHTLQASLGCEGREDPMAWGLKEKWEADLNRGDRDRTFLEGT*THMLRKYLASGGKPGESXX

VPPTVQLTSHLGPSRETILKCWALGFSPWNISLTGCRDGETLPQGTLEPEVGLPSGDGTYQNWGVLRVSPGAGQRCTCHADHQGLARPLTVPW

>Pou5f1

MAGHLGPDFAFSPPPGGGEPWVDPRGWYGYPAPPGAAGAGAGAGAGGEPWGGPFLPPPAG

PLYEGGGWAGGCFVPQIGVGLAQPPPDPSGPDGGGRGGRRDAPPGRLPRAGGLRAGPEAG

AGRERRRASPPRPRPQQETPSREELEQFAKELKRKRITLGYTQADVGVTLGALFGKVFSQ

TTICRFEAQQLSFKNMCKLRPLLQRWLEAADDNDRLQEMCNAETVLQQARKRKRTSIENK

VRGNLETMFLQCPKPNLQQISSIAEELGLEKDVVRVWFCNRRQKGKRGSGCSSREDFETA

GPFPSGPVHLPLPPGPSLGPPGYGGPPFATLYSPAPFHDGDAFAPVPIAPLGPPTHSS*

>Tcf19

MLPCFQLLRMGGGGGGDLYTFRPAGTGCTYRLGRRADLCDVPLRSEREPGRVSRVHAELH

AERDAQRDGDWRVSLVDCSSHGTSVNDVRLPRGRRVELDDGDLLTFGSEGGPGAEPPEFC

FMFQRVRVRPQDFAAITVPRAVGVGGGFRPMLPSRGAPQRPLSAPSPSPKATLILSSIGS

LSKLRPQPLTFSRGGGPVEAPAPAPAVGGKGAPDVPPAPRNRRKSAHRVLAELEDEGVPG

ESPPAARPGPRKKPRMERTPVTPGGKRRGRPRKYPIAPVPGGDPCAAPRCRLPQDEMVTW

VQCDHCDAWFHVACAGCSYRAAQEADFRCPTCRA*

>Hcr

MASRRWNLDPPAAFGVTQAAGSRELVPPSHFQARPPAAPSWARAGVQLEVLELRRENQQL

RAAMWGWGGGRAAAEDPGSGARFRDQERSQGLSQQAQLVSPQPQELPRLEAEAQQGAKLE

AQAEELEALGRAEVEELRASLARAEEARLRLEEGGQQELEAARRQHEEEVAHLTQAHLEA

LSTLRGQVQELHETVQELEARRGQEAMALAAAQAEADTLQEQLKKSREELEARDSLVRQL

RRHVSEQGVAQSRCQAWEQERGELQQAVKRLQEERAALSSTSELLQVRVQSLTDILTLQE

RELAQKAPNVNILEPESARKARALLSRWREKVFSLMVQLKAQELSHAKHTCQLQKEVAEL

EKEVASQRQQEALLLHSLKGKEAEIQMEQVNSKVLQGELSRSQDSKQQLQDQVQAAEEQV

QLLSESVHSSQQGLLARMAEVEVALAQLPGLSTRLSYAARQVRILQGLVARKVALAQIRQ

EESAPAPAEAEALDTELRQLREERSRQDAELRFSARLLQQEVARARDQGREERQRLAEAS

QRLERELRESREALGEAEARLEAALAGQRDSQDQAASLRRELGKQQEVYERVLQEKVSEV

EARLREPLAALEKELSAARREHSKAVVSLRQVQRQAARDRERNLELRRLHEEARQEQEAR

TSRRLQQLERDKHLLLVRTLPSSPSHRPISLPIPFPGRPGQAGSYTHPHTSPPPPPTPEG

M*

>Spr1

MLDFKLLGILVLCLHAGGITGSGDPPPQPPADAPEEAAAPTWPQGPPIPGDPWPGVPPIF

EDPPPPGPSRRSLPEPGVWPPNPGRPSTPQPPRPDDPWPAGPQPPENPWPPGPEMDAAPQ

HEPDFDPPREEYR*

>Cdsn

MGRARGPRSGLVGGHTVLLLLVIGLLLPGILSKSISSVLDPCKDPSRISSPALAALCKPR

IGSSSGSSGSSGSSGSSGSSGSSGSSGSSGSSGSSGSSGSSGSSGSSGSSGTSGSSGSSG

SSGSSGSGKEWSSQTGTSWSSQWGTGSSQSNAGWPVQSGTGSSQSSAGWPVHGTGAYQPG

PGSSYPSSEWPLQWGDLSSSDGSQTAWLSSSSSSSSSSGGGGGGGIRGVALYGPCSSGSG

PSSVPCNAPSSYLPDSHTVAGGQRPVVVVETHGGPCAPGRPCPPITSLGGGPAGGGYEVV

GGSAQDYLVPGMTFDKGKIYPVGYFTKEQGPKGAAGRPVFAAGAPVSEGVYFSSNAYPAQ

HSAGALPSPAHTGLFSQTGGALIAFRAPGSPHGGCGAGSRGGPCSPSFSSSSSSSSSSSS

SSAGALQPCGGRGSQGSQGSGSACPPAGSRGSSGGSPGGPVLVVQSCGSGGGGSPGRPCG

SSPPGLSGGPDGSPHPDPSAGAKPCGSGARGSSGLPCRSIRDIWAQVKPLGPQLVNPAVF

LPQGDPLRAP*
