# Supplementary material for: Disruption and pseudoautosomal localization of the major histocompatibility complex in monotremes
Source: Genome Biol. 2007 Aug 29;8(8):R175. doi: 10.1186/gb-2007-8-8-r175 (PMC2375005; doi:10.1186/gb-2007-8-8-r175)
Supplement: Additional data file 10 — Accession numbers for MHC class II genes used for phylogenetic analysis in Figure 3. [file gb-2007-8-8-r175-S10.doc]

SUPPL. TABLE 4a

Accession numbers for Class II genes used for phylogenetic analysis in Fig. 3
