# Supplementary material for: Disruption and pseudoautosomal localization of the major histocompatibility complex in monotremes
Source: Genome Biol. 2007 Aug 29;8(8):R175. doi: 10.1186/gb-2007-8-8-r175 (PMC2375005; doi:10.1186/gb-2007-8-8-r175)
Supplement: Additional data file 11 — Accession numbers for MHC class I genes used for phylogenetic analysis in Figure 4. [file gb-2007-8-8-r175-S11.doc]

SUPPL. TABLE 4b

Accession numbers for Class I genes used for phylogenetic analysis in Fig. 4
